# Clinical, cytological and ultrasonographic features of incidental thyroid cancer in a hospital‐based study in vietnam

**DOI:** 10.1002/edm2.415

**Published:** 2023-03-10

**Authors:** Bay Quang Nguyen, Hau Thi Tran, Huong Thi Thu Nguyen, Thanh Xuan Nguyen, Thu Thi Hoai Nguyen, Tam Ngoc Nguyen, Huyen Thi Thanh Vu

**Affiliations:** ^1^ Internal Medicine Department Hanoi Medical University Hanoi Vietnam; ^2^ Department of Endocrinology Bach Mai Hospital Hanoi Vietnam; ^3^ National Hospital of Endocrinology Hanoi Vietnam; ^4^ Department of Geriatrics Hanoi Medical University Hanoi Vietnam; ^5^ Scientific Research Department National Geriatric Hospital Hanoi Vietnam

**Keywords:** incidental, thyroid cancer, thyroid nodules FNAB

## Abstract

**Introduction:**

Thyroid nodules are common diseases of the endocrine system, with a 5% prevalence rate in the general population. This study aimed to identify prevalence, clinical, cytological and ultrasonographic features of incidental thyroid cancer and its associated factors in Vietnam.

**Methods:**

This cross‐sectional descriptive study consisted of 208 patients with incidental thyroid nodules detected by ultrasound at the Endocrinology Department, Bach Mai Hospital, Hanoi, Vietnam between November 2019 and August 2020. Clinical information, sonography characteristics of thyroid nodules, results of fine‐needle aspiration biopsy (FNAB), postoperative pathology and lymph node metastasis were collected. A multiple logistic regression model was used to estimate factors associated with thyroid cancer.

**Results:**

A total of 272 thyroid nodules (from 208 participants) were included in this study. The mean age was 47.2 ± 12.0 (years). The rate of incidental thyroid cancer patients detected was 17.3%. Nodules <1 cm in size were significantly more prevalent for malignant nodules. The size of more than half of thyroid cancer nodules was 0.50–0.99 cm. Postoperative pathology of all nodules with Bethesda V and VI was papillary thyroid cancer which was consistent with cytological results. 33.3% of thyroid cancer patients have lymph node metastasis. The regression model showed that thyroid cancer was more likely to occur at a younger age (≤ 45 years vs. >45 years, OR 2.8; 95% CI: 1.3–6.1), taller‐than‐wide nodules (OR 6.8; 95% CI: 2.3–20.2) and hypo‐echoic nodules (OR 5.2; 95% CI: 1.7–15.9).

**Conclusion:**

The study showed that the prevalence of incidental thyroid cancers was 17.3%, of which 100% was papillary carcinoma. People under the age of 45 and the presence of ultrasound characteristics, such as taller‐than‐wide and hypoechoic nodules increased risk for malignancy.

## Graphical abstract

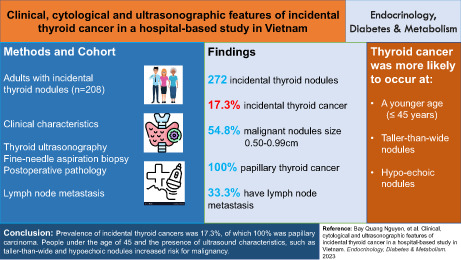

## 1 INTRODUCTION

Thyroid nodules are common diseases of the endocrine system, with a 5% prevalence rate in the general population.^1^ Although 85%–93% of thyroid nodules are benign, they may become potentially malignant, which has become the fastest‐growing cancer. A thyroid incidentaloma is an unexpected, asymptomatic thyroid nodule discovered incidentally during examination for an unrelated condition. The incidence of thyroid incidentaloma varies depending on different imaging modalities. Previous studies reported that the rate of thyroid incidentaloma was 67% with neck ultrasonography imaging,^2^ 0.84% of carotid ultrasonography,^3^ 15% with computed tomography (CT) or magnetic resonance imaging (MRI) of the neck,^4^, ^5^ 3.45% of cervical spine or face/orbit MRIs, 5.84% of chest CTs with contrast, and 5.14% of chest CTs without contrast,^3^ 1%–2% with fluorodeoxyglucose (FDG) positron emission tomography^6^, ^7^ and 5% of time‐resolved magnetic resonance angiography (TRMRA) examinations.^8^ Vietnam has witnessed a rapid increase in the incidence of thyroid cancer. The age‐standardized incidence rate of thyroid cancer was 2.4 per 100,000 during 1996–2000 and dramatically increased to 7.5 per 100,000 during 2011–2015.^9^

With the development of imaging technologies, the probability that asymptomatic thyroid nodules will be discovered is increasing. In the absence of accurate clinical signs or predictors of malignancy, many of the nodules will require fine‐needle aspiration biopsy (FNABs), which appears not to be cost‐effective and diagnostic challenges, for example indeterminate and non‐diagnostic cytology. Clinical evaluations and sonographic variables should be used in combination to determine whether the clinician should proceed with further confirmatory tests or with periodical follow‐up.^10^, ^11^ High‐risk clinical features such as young age, single nodularity and male sex were established risk factors for malignant thyroid nodules.^12^ The ultrasound findings of micro‐calcifications, absence of a halo, hypo‐echogenicity and irregular nodular margins all increase cancer risk^13^, ^14^

The 5‐year survival rates for thyroid cancers are nearly 100% for localized tumours, 97% for tumours with regional metastases and 57.3% for distant staged tumours.^15^ Early detection of thyroid cancers can have a significant positive effect on prognosis and impact treatment decisions.^16^ The aim of this study was to describe the prevalence, clinical, cytological and ultrasonographic features of incidental thyroid nodules and to assess the independent predictors of thyroid cancer in Vietnam.

## 2 MATERIALS AND METHODS

### 2.1 Study design and setting

The study was designed as a cross‐sectional investigation including 208 subjects with incidental thyroid nodules, who presented to the Endocrinology Department, Bach Mai Hospital, Hanoi, Vietnam between November 2019 and August 2020.

### 2.2 Subjects

#### 2.2.1 Inclusion criteria

All subjects had incidental thyroid nodules detected by thyroid ultrasound during routine check‐ups. They had no history, palpable or other clinical signs of thyroid disease and agreed to participate in this study.

#### 2.2.2 Exclusion criteria

Participants were excluded from the study if they had any of the following conditions: (1) a history of thyroid diseases (e.g. thyroid nodules, Grave's disease, hypothyroidism, thyroiditis, thyroidectomy, ectopic thyroid gland and thyroglossal duct cyst) (2) known primary cancer of other organs, (3) symptomatic thyroid nodules (e.g. dysphagia, globes sensation, hoarseness, pain, hyperthyroidism syndrome and hypothyroidism syndrome) (4) missing laboratory results (e.g. FT4, TSH, thyroid ultrasound and cytology).

The sample size was calculated using a single population proportion formula: *n* = *Z* ^2^_1‐ α/2_ * [p*(1‐p)/d^2^], with n = the required sample size, *Z*_1‐ α/2_ = 1.96 (with *α* = .05 and 95% confidence interval) and d = precision (assumed as 0.05). With *p* = .15 (according to proportion of patients with incidental thyroid cancer of a previous study by Jin J et al.),^17^ we found the essential sample size to be 196 participants. To compensate for the subjects who have lost follow‐up after surgery or may end up refusing to participate, an additional 5% was added to the sample size. Eligible subjects were recruited by convenience sampling until the final sample size of 206 was reached. All subjects without sufficient information were excluded from the final data. Data from a total of 208 patients were used for analysis.

The study was approved by the Ethics Committee of the Bach Mai Hospital Review Board, (Reference Number: 837/BVBM‐HDDD). Written informed consent was obtained from all participants. This study complied with the Declaration of Helsinki.

### 2.3 Data collection

#### 2.3.1 Clinical data

A questionnaire was designed to collect information from participants, including age, gender, past history (including radiation exposure, thyroid diseases), family history (thyroid diseases, multiple endocrine neoplasia (MEN), familial adenomatous polyposis (FAP), Gardner syndrome, Cowden syndrome, Carney). A resident doctor was trained to collect the data.

#### 2.3.2 Subclinical data

##### * Blood tests

All participants were taken blood to measure FT4 and TSH by Electrochemiluminescence immunoassay (ECLIA) at the Department of Biochemistry, Bach Mai hospital. The normal range of FT4 and TSH is 12–22 pmoL/L and 0.27–4.2 μU/ml, respectively. Thyroid function was classified according to ATA 2016.^18^

* **Thyroid ultrasonography** was conducted with a Korean Samsung Medison ultrasound system (Model UGEO G60 ‐ USS‐G60NF40/WR) with a 7,5 MHz probe. The ultrasound technique was standardized to include transverse and longitudinal images of both lobes of the thyroid gland as well as the adjacent structures in the neck. All nodules were measured in 3 planes, and hard copy images were created to document nodule location and orientation. Thyroid nodules were characterized by the number, location, echogenicity, margin and shape of nodules. Thyroid nodules were classified according to the American College of Radiology Thyroid Imaging Reporting and Data Systems (ACR‐TIRADS) 2017.^19^ The nodule size is classified according to the largest diameter of the thyroid nodule. According to the position, the thyroid nodules were subdivided into the following three groups: left lobe, right lobe and isthmus.

* **Ultrasound‐guided fine‐needle aspiration (US‐guided FNA)** was performed with a 25‐gauge needle and a 5‐mL disposable syringe. The sterile gel was used as the coupling agent, and the needle was inserted along a path within the scanning plane so that the nodule and the needle were continuously visualized. When the needle reached the target, aspiration biopsy was performed with an in‐and‐out movement while suction was applied. Suction was released before the needle was removed from the nodule. The collected material was expelled onto glass slides, fixed immediately by a trained cytotechnologist and placed into a CytoLyt solution for centrifugation. FNA results were interpreted by pathologists at the Pathology Center, Bach Mai Hospital, and classified by Bethesda 2018.^20^ Uncertain findings would be reviewed by experienced specialists. Based on the results of the FNA biopsy and thyroid function test, endocrinologists would give appropriate management decisions for patients.

The thyroid ultrasound and US‐guided FNA protocols were developed, standardized and trained for three experienced and well‐trained endocrinologists at Endocrinology Department, Bach Mai Hospital. After training, these physicians were re‐tested before starting the study to confirm the results were similar. Each patient had an ultrasound and FNA biopsy by one of these three endocrinologists.

##### * Pathology results after surgery

All patients with Bethesda V and VI were performed thyroidectomy in three hospitals, which were Bach Mai Hospital, Vietnam National Cancer Hospital and National Hospital of Endocrinology. The postoperative pathology results were evaluated using the WHO 2017 classification.^21^

### 2.4 Statistical methods

The analysis was performed with SPSS 20. Descriptive statistics were used: continuous variables were presented as mean ± standard deviation (SD) and categorical variables as frequency and percentage. Proportions were compared by using Chi‐squared tests with continuity correction or Fisher's exact test when appropriate, continuous variables were compared by using Student's *t*‐tests. Univariate logistic regression was performed on general factors and other potential factors that can be associated with thyroid cancer based on the literature such as the size of a thyroid nodule, and ultrasonographic features. Only variables that had a *p*‐value <.2 on univariate analysis were selected for multivariate analysis. Multivariate analyses were conducted with logistic regression, the results were presented as an odds ratio (OR) with a 95% confidence interval (CI). All values of p < .05 were considered statistically significant.

## 3 RESULTS

A total of 272 thyroid nodules (from 208 participants) were included in this study. The mean age was 47.2 ± 12.0 (years). The proportion of patients with thyroid cancer detected was 17.3%. The rate of thyroid cancer in the age group ≤45 (years) was statistically significantly higher than that in the age group >45 (years) (Table 1).

**TABLE 1:** General characteristics of participants.

Characteristics		Total (*n* = 208 patients)		Cancer (*n* = 36 patients)		Non‐cancer (*n* = 172 patients)		*p*‐Value
		*N*	%	*n*	%	*n*	%	
All		208	100	36	17.3	172	82.7	
Gender	Female	181	87.0	32	88.9	149	86.6	.714
	Male	27	13.0	4	11.1	23	13.4	
Family history of thyroid cancer	Yes	2	1.0	1	2,8	1	0,6	>.05
	No	206	99.0	35	97,2	171	99,4	
Age groups	≤45	96	46.2	25	69.4	71	41.3	.003
	>45	112	53.8	11	30.6	101	58.7	
TSH	Normal	200	96.2	36	100	164	95.3	.419
	Low	5	2.4	0	0	5	2.9	
	High	3	1.4	0	0	3	1.7	
		Mean ± SD		Mean ± SD		Mean ± SD		
Age (year) Min ‐ Max		47.2 ± 12.0 13–81		42.2 ± 12.0 16–73		48.3 ± 11.8 13–81		.006
Mean TSH (μU/ml)		1.69 ± 1.0		1.67 ± 0.65		1.7 ± 1.06		>.05
Mean FT4 (pmol/l)		12.82 ± 2.66		12.55 ± 2.7		12.88 ± 2.65		>.05

The sonographic characteristics of those with cancer versus non‐cancer ones were compared in Table 2. About nodule sizes, those with sizes <1 cm were significantly more prevalent for a malignant nodule (*p* < .05). More than half of thyroid cancer nodules were 0.50–0.99 cm in diameters. Malignant nodules were more likely to be solid (92.9% vs. 58.7%, *p* < .001), taller‐than‐wide (33.3% vs. 3%), hypo‐echoic (92.9 vs. 48.7%, *p* < .001), microcalcification (21.4% vs. 7%, *p* = .003) and blurred margins (50.0% vs. 15.7%, *p* < .001) nodules.

**TABLE 2:** Ultrasonographic characteristics of incidental thyroid nodules.

Characteristics		Total *N* = 272 nods		Cancer *n* = 42 nods		Non‐cancer *n* = 230 nods		*p*‐Value
		*N*	%	*n*	%	*n*	%	
Type of thyroid nodules	Solitary	98	47.1	16	44.4	82	47.7	.855
	Multinodular	110	52.9	20	55.6	90	52.3	
Number of thyroid nodules		1.88 ± 1.05		1.92 ± 1.08		1.87 ± 1.05		>.05
Size of thyroid nodule (cm)	< 0.50	18	6.6	8	19.0	10	4.3	<.001
	0.50–0.99	117	43.0	23	54.8	94	40.9	
	1.00–1.49	85	31.3	4	9.5	81	35.2	
	≥1.50	52	19.1	7	16.7	45	19.6	
Thyroid lobe	Left	116	42.7	13	31.0	103	44.8	>.05
	Right	145	53.3	26	61.9	119	51.7	
	Isthmus	11	4.0	3	7.1	8	3.5	
Ultrasonographic features								
Echo structure	Solid	174	64.0	39	92.9	135	58.7	<.001
	Cystic	13	4.8	0	0	13	5.7	
	Mixed	85	31.2	3	7.1	82	35.6	
Taller‐than‐wide	Yes	21	7.7	14	33.3	7	3.0	<.001
	No	251	92.3	28	66.7	223	97.0	
Echogenicity	Hypoechoic	151	55.5	39	92.9	112	48.7	<.001
	Isoechoic	26	9.6	0	0	26	11.3	
	Hyperechoic	5	1.8	0	0	5	2.2	
	Mixed	77	28.3	3	7.1	74	32.2	
	Non‐echoic	13	4.8	0	0	13	5.6	
Microcalcification	Present	25	9.2	9	21.4	16	7.0	0.003
	Absent	247	90.8	33	78.6	332	93.0	
Margins	Blurred	57	21.0	21	50.0	36	15.7	<.001
	Well‐defined	215	79.0	21	50.0	194	84.3	

Cytological characteristics of FNA, postoperative pathological and lymph node metastasis are shown in Table 3. The FNAB results found that 74.3% of nodules were benign cytology (Bethesda II), 1.5% were follicular neoplasm (Bethesda IV), and 4% were suspicious of malignancy (Bethesda V) and 13.2% were malignant cytology (Bethesda VI). The postoperative pathology of all patients with Bethesda V and VI was papillary cancer.

**TABLE 3:** Bethesda classification and histopathological results after surgery.

Cytological diagnosis Bethesda classification	FNAB n (%)	Surgical nodule *n* (%)	Cancer *n* (%)	Non‐Cancer *n* (%)	Type of cancer *n* (%)	Lymph node metastasis *n* (%)
Nondiagnostic I	19 (7.0)	0	0	0		
Benign II	202 (74.3)	0	0	0		
AUS/FLUS III	0	0	0	0		
Follicular neoplasm IV	4 (1.5)	0	0	0		
Suspicious of malignancy V	11 (4.0)	9 (21.4)	9 (21.4)	0	Papillary carcinoma 9(21.4)	3 (21.4)
Malignancy VI	36 (13.2)	33 (78.6)	33 (78.6)	0	Papillary carcinoma 33(78.6)	11 (78.6)
Total nodules	272	42	42		42	14 (33.3%)

*Note*: three patients (5 nodules) with Bethesda V and VI refused surgery.

Of those having surgery, 33.3% of thyroid cancer patients had lymph node metastasis. According to the AJCC 8th classification of differentiated thyroid cancer, there are 35 patients with stage I, and 1 patient with stage II.

On multiple logistic regression analysis (Table 4), thyroid cancer was more likely to occur at a younger age (≤45 years vs. >45 years, OR 2.8; 95% CI: 1.3–6.1), taller than wide nodules (OR 6.8; 95% CI: 2.3–20.2), hypo‐echoic nodules (OR 5.2; 95% CI: 1.7–15.9).

**TABLE 4:** Multiple logistic regression model assessing independent predictors of thyroid cancer.

	Thyroid cancer				
	Univariate			Multivariate	
	OR	95% CI	*p*	OR	95% CI
Age ≤45 (Ref: >45 years)	2.6	1.2–5.8	.018	2.8*	1.3 ‐ 6.1
Gender Female (Ref: male)	1.0	0.3–3.2	.578	‐	‐
Size of thyroid nodule <1 cm (Ref: ≥1 cm)	1.2	0.5–2.8	.738	‐	‐
Echo structure Solid (Ref: No‐solid)	1.7	0.3–9.3	.883	‐	‐
Taller‐than‐wide Yes (Ref: No)	6.4	2.1–19.3	.000	6.8*	2.3 ‐ 20.2
Echogenicity Hypo‐echoic (Ref: Non‐hypoechoic)	3.6	0.8–15.8	.030	5.2*	1.7 ‐ 15.9
Micro‐calcification Present (Ref: Absent)	1.1	0.3–3.5	.769	‐	‐
Margins Blurred (Ref: Well‐defined)	2.1	0.9–5.0	.038	2.1	0.9–4.9

* *p* < .05.

## 4 DISCUSSION

To our knowledge, the current study is the first work to mention incidental thyroid nodules in a hospital‐based study in Vietnam. The results showed that the rate of thyroid cancer was 17.3% out of 208 patients with thyroid nodules detected incidentally on ultrasound. Thyroid cancer patients were mostly aged ≤45 and most of the nodules with diameter ranged from 0.50 to 0.99 cm. The sonographic features of nodules associated with a high risk of malignancy were those with taller‐than‐wide dimensions and hypo‐echogenicity.

The incidence of malignancy in incidental thyroid nodules varies depending on the diagnosis technique being computed tomography (CT)/magnetic resonance imaging (MRI), ultrasound or positron emission tomography (PET), ranging from 5% to 13%,^7^, ^22^ 12% to 21,6%^23^, ^24^, ^25^ and up to 33%,^26^ respectively. The cancer rate in our study was similar to the results of Liebeskind A. et al^23^ showing that the malignancy rate of FNA results in incidentally discovered nodules on ultrasound was 17%, which was a higher rate of cancer detected by CT/MRI and a lower rate by PET. This may be explained by the fact that CT/MRI investigations do not have a specific criterion like the TIRADS grading in ultrasound for characterizing a benign or malignant thyroid nodule and the Fluorodeoxyglucose test‐PET is often performed in the setting of another malignancy.

Age is an important prognostic factor of differentiated thyroid cancer as outlined in classification systems such as TNM, AMES and MACIS.^27^, ^28^, ^29^ On multiple logistic regression analysis in our study, thyroid cancers were more likely to occur at a younger age (≤45 years vs. >45 years, OR 2.7, 95% CI 1.2–6.1). In Vietnam, according to a study by Dung X. Pham et al. about the incidence and histological pattern of thyroid cancer in Ho Chi Minh City from 1996 to 2015, the incidence of thyroid cancer was highest at the age of <45 in both genders.^9^ It is also in concurrence with the findings of Chen et al. in the United States, a study of differentiated thyroid cancer diagnosed in all SEER registries from 1988 to 2005, the majority of cases occurring in Caucasians (83%), women (76%) and individuals <45 years old (48%).^30^

Many studies have attempted to show that certain sonographic features are associated with a higher risk of malignancy, especially in nonpalpable thyroid nodules to avoid unnecessary FNA or surgery.^31^, ^32^, ^33^ In our study, the ultrasonographic features of nonpalpable nodules suggesting the highest malignancy odds ratios were taller‐than‐wide dimensions (OR 6.8, 95% CI 2.3–20.2) and hypo‐echogenicity (OR 5.2, 95% CI 1.7–15.9). Meanwhile, a study by Kang HW et al.^32^ indicated that the ultrasound features of solid echogenic structures, blurred margins and calcifications showed significant diagnostic value in detecting malignancy in incidental thyroid nodules. In fact, blurred margin, calcification, hypo‐echogenicity and taller‐than‐wide and solid echo structures are all suggestive of cancer and have a high TIRADS score.^18^ However, in small thyroid nodules, ultrasonography may be more difficult to detect features such as calcifications and blurred margins than taller‐than‐wide and hypoechoic.

Nonpalpable thyroid nodules are usually small in size. According to American Thyroid Association 2015, FNA is recommended for those nodules ≥1 cm and TIRADS 4, 5.^33^ However, our data showed that the incidentally detected thyroid cancer was mostly in the size from 0.5 to 0.99 cm (54.8%) and <0,5 cm (19.0%). In the United States, in the period 1988–2005 the highest rate of increase was for primary tumours <1.0 cm.^30^ Additionally, in Zhejiang Province, South East China, most of the increased incidence was papillary thyroid carcinoma, especially the papillary thyroid microcarcinoma with maximum tumour diameter <1 cm. The proportions of patients increased from 0 in 1972–1985 to 32.1% in 2000–2014.^34^ This increase resulted from expanded thyroid ultrasound screening and ultrasound guide fine‐needle aspiration biopsies. Therefore, clinicians should recommend ultrasound‐guided FNA to screen for malignancy for incidentally detected thyroid nodules, even when the nodule size is <1 cm, with sonography features suspicious on such as taller‐than‐wide dimensions and hypo‐echogenicity in patients ≤45 years of age. Our result had a majority of women (87%) who are at the highest risk of developing thyroid cancer earlier (those were most often in their 40s or 50s at diagnosis) than men (often in their 60s or 70s).^35^ This finding may also be related to the role of oestrogen levels, its receptors and gynaecological diseases requiring oestrogen replacement therapy in the proliferation, migration and invasion of thyroid cancer.^36^, ^37^, ^38^ Women >45 years of age are often perimenopausal or postmenopausal and have declining oestrogen levels which may be associated with a lower incidence of thyroid cancer^39^, ^40^, ^41^, ^42^ (the average age of menopause in Vietnam has been reported to be 48 or 49 years^43^). However, more research is needed to clarify about these associations. In addition, it may be because people <45 years of age are the beneficiaries of routine physical examination including thyroid ultrasound.

In our study, cytological and histopathology results were 100% papillary thyroid carcinoma (PTC), possibly due to the size of nodules and ethnicity. Pamela L. et^44^ investigated how birthplace influences the incidence of PTC among Asian American women, the results show that Vietnamese and Filipina women experienced incidence rates that were significantly greater than women in the other three Asian ethnic groups and non‐Hispanic white women (8.01 per 100,000). The difference may be due to the immigration and acculturation of these Asian ethnic groups (e.g. goitrogenic exposure, diet, weight and body mass index and menstruation and reproductive events), which can have a strong effect on the development of PTC. In terms of diet, Iodine deficiency is thought to be associated with the follicular and anaplastic histotypes whereas iodine excess is related to the papillary histotype.^45^ Molecular alterations (including the BRAF mutations and rearrangements) were identified mainly in PTC but rare in the follicular thyroid cancer (FTC).^46^, ^47^ Previous studies showed that the percentage of BRAF‐positive PTCs in iodine‐sufficient areas was higher than in iodine‐deficient areas.^48^, ^49^ Vietnam is a country with a long coastline and the national program to prevent Iodine deficiency disorders has been implemented since 1993, which can lead to iodine excess associated with the predominance of papillary thyroid cancer in our study.^50^ It should be noted that our study has not found any follicular thyroid cancer. Interestingly, the FTC exhibits other differences in sonographic features compared to those in the PTC. The latter is more likely to be iso‐ to hyperechoic, noncalcified, wider‐than‐tall nodules with well‐defined margins.^51^ In contrast, the results in our study with PTC nodules had the ultrasound features of hypo‐echogenicity, micro‐calcifications, taller‐than‐wide dimensions and blurred margins. In addition, in the study of Al‐Hakami H et al,^52^ follicular carcinoma was mostly found in nodules with a size of 4 cm, on the other hand, the largest nodules in our study were 2.4 cm.

There are some strengths and limitations to our study. This has been the first study in Vietnam to discover random thyroid nodules. We found it possible to use ultrasound—a cost‐effective and easily accessible tool—to detect thyroid cancer with great accuracy and relevance in developing countries. However, this was a cross‐sectional study that could not show a causal relationship between risk factors and outcomes. Therefore, it is essential for further studies to explore these issues.

## 5 CONCLUSION

In conclusion, the current study emphasizes that incidental thyroid cancers were not uncommon with a prevalence of 17.3%, in which 100% was papillary carcinoma. People under the age of 45 and the presence of ultrasound characteristics, such as taller‐than‐wide and hypoechoic nodules increased risk for malignancy. Ultrasound imaging could be used to assist in the clinical risk assessment of thyroid cancer.

## AUTHOR CONTRIBUTIONS

**Bay Quang Nguyen:** Conceptualization (equal); data curation (equal); formal analysis (equal); investigation (equal); methodology (equal); project administration (equal); resources (equal); software (equal); supervision (equal); validation (equal); visualization (equal); writing – original draft (equal); writing – review and editing (equal). **Hau Thi Tran:** Conceptualization (equal); data curation (equal); formal analysis (equal); investigation (equal); methodology (equal); project administration (equal); resources (equal); software (equal); supervision (equal); validation (equal); visualization (equal); writing – original draft (equal); writing – review and editing (equal). **Huong Thi Thu Nguyen:** Conceptualization (equal); data curation (equal); formal analysis (equal); investigation (equal); methodology (equal); project administration (equal); resources (equal); software (equal); validation (equal); visualization (equal); writing – original draft (equal); writing – review and editing (equal). **Thanh Xuan Nguyen:** Conceptualization (equal); data curation (equal); formal analysis (equal); investigation (equal); methodology (equal); project administration (equal); resources (equal); software (equal); supervision (equal); validation (equal); visualization (equal); writing – original draft (equal); writing – review and editing (equal). **Thu Thi Hoai Nguyen:** Conceptualization (equal); data curation (equal); formal analysis (equal); investigation (equal); methodology (equal); project administration (equal); resources (equal); software (equal); supervision (equal); validation (equal); visualization (equal); writing – original draft (equal); writing – review and editing (equal). **Tam Ngoc Nguyen:** Conceptualization (equal); data curation (equal); formal analysis (equal); investigation (equal); methodology (equal); project administration (equal); resources (equal); software (equal); supervision (equal); validation (equal); visualization (equal); writing – original draft (equal); writing – review and editing (equal). **Huyen Thi Thanh Vu:** Conceptualization (equal); data curation (equal); formal analysis (equal); funding acquisition (equal); investigation (equal); methodology (equal); project administration (equal); resources (equal); software (equal); supervision (equal); validation (equal); visualization (equal); writing – original draft (equal); writing – review and editing (equal).

## FUNDING STATEMENT

This research did not receive any specific grant from funding agencies in the public, commercial, or not‐for‐profit sectors

## CONFLICT OF INTEREST STATEMENT

The author reports no conflicts of interest in this work.

## Data Availability

The datasets of this study are available from the corresponding author on reasonable request.
